# Identification of Potential RBPJ-Specific Inhibitors for Blocking Notch Signaling in Breast Cancer Using a Drug Repurposing Strategy

**DOI:** 10.3390/ph15050556

**Published:** 2022-04-29

**Authors:** Mengjie Rui, Min Cai, Yu Zhou, Wen Zhang, Lianglai Gao, Ke Mi, Wei Ji, Dan Wang, Chunlai Feng

**Affiliations:** Department of Pharmaceutics, School of Pharmacy, Jiangsu University, Zhenjiang 212013, China; mjrui@ujs.edu.cn (M.R.); 2211915001@stmail.ujs.edu.cn (M.C.); 2221815020@stmail.ujs.edu.cn (Y.Z.); 2221915025@stmail.ujs.edu.cn (W.Z.); 2222015007@stmail.ujs.edu.cn (L.G.); 2212015010@stmail.ujs.edu.cn (K.M.); jinjian@ujs.edu.cn (W.J.); danwang@ujs.edu.cn (D.W.)

**Keywords:** Notch signaling, RBPJ protein, drug repurposing, fidaxomicin, acarbose, schaftoside, breast cancer

## Abstract

Notch signaling is a key parameter in regulating cell fate during tissue homeostasis, and an aberrant Notch pathway can result in mammary gland carcinoma and has been associated with poor breast cancer diagnosis. Although inhibiting Notch signaling would be advantageous in the treatment of breast cancer, the currently available Notch inhibitors have a variety of side effects and their clinical trials have been discontinued. Thus, in search of a more effective and safer Notch inhibitor, inhibiting recombinant signal binding protein for immunoglobin kappaJ region (RBPJ) specifically makes sense, as RBPJ forms a transcriptional complex that activates Notch signaling. From our established database of more than 10,527 compounds, a drug repurposing strategy-combined docking study and molecular dynamic simulation were used to identify novel RBPJ-specific inhibitors. The compounds with the best performance were examined using an in vitro cellular assay and an in vivo anticancer investigation. Finally, an FDA-approved antibiotic, fidaxomicin, was identified as a potential RBPJ inhibitor, and its ability to block RBPJ-dependent transcription and thereby inhibit breast cancer growth was experimentally verified. Our study demonstrated that fidaxomicin suppressed Notch signaling and may be repurposed for the treatment of breast cancer.

## 1. Introduction

The Notch signaling pathway is highly studied and is responsible for cellular communication and cell fate decision throughout a wide variety of developmental processes [1,2,3]. Notch signaling is initiated by ligand binding, followed by a series of cleavage events and the induced release of an intracellular domain (NICD). NICD then directly translocates into the cell nucleus and interacts with a transcription factor RBPJ (recombinant signal binding protein for immunoglobin kappaJ region) [4,5,6]. The resultant NICD/RBPJ complex further recruits transcriptional coactivators to activate the expression of Notch downstream target genes such as Hes1, Hes5, and Hey1 [7,8]. Aberrant activation of Notch signaling, which plays a critical role in cellular development, is linked to a number of diseases, including Alzheimer’s disease, ischemic stroke, heart disease, and cancer [9,10,11,12]. Given its tumor-promoting role, selective inhibition of the Notch signaling pathway represents a valuable therapeutic target in cancer therapy.

A large number of Notch inhibitor candidates have been designed to inhibit γ-secretase, which mediates the cleavage of Notch receptors to liberate NICD. For example, a well-known small peptide inhibitor called DAPT {N-[N-(3,5-difluor-ophenacetyl)-l-alanyl]-S-phenylglycine tert-butyl ester} effectively inhibits the activity of γ-secretase (Supplementary Material Figure S1) [13]. These γ-secretase inhibitors developed in recent years have been found to cause a variety of toxic effects, including gastrointestinal bleeding, immunosuppression, cancerous skin lesions, and cognitive worsening in Alzheimer’s Disease patients [14,15]. Therefore, more than half of the clinical trials of γ-secretase inhibitors have been halted [16,17]. Notch inhibitors should be further developed to avoid adverse effects, with an emphasis on increasing Notch selectivity. Another intriguing target in Notch signaling is the RBPJ protein, which is involved in both transcriptional activation and repression of Notch signaling [6,18]. Following its interaction with NICD and other coactivators, the activity of RBPJ shifts from repressing transcription to activating the transcription of Notch downstream genes. The rationale for developing an RBPJ inhibitor lies in the promise that disrupting the formation of the NICD–RBPJ–DNA complex provides a more specific means of modulating Notch function than γ-secretase inhibition.

The application of structure-based drug design in the search for novel RBPJ inhibitors benefits the translation of new drugs to the market. However, in spite of previous reports of novel RBPJ inhibitors [19,20], none has yet made significant progress clinically. RBPJ’s structure indicates that it has various binding sites that are open for other molecules, including NICD, coactivators, and DNA [21,22,23]. As a result, small molecules are likely to occupy one of the RBPJ’s binding sites to inhibit the formation of a transcriptional complex. It is theoretically possible to repurpose existing approved and investigational drugs as RBPJ inhibitors. Compared to de novo drug design, drug repurposing has numerous advantages: lower risk, reduced cost, and a shorter time to market [24,25,26].

In this study, we established a structure-based virtual screening strategy for identifying potential RBPJ-specific inhibitors from a database that collected compounds from a variety of sources. Following a molecular docking-based first-round screening, we performed molecular dynamic simulations to verify the hits and determine their binding free energies. Moreover, we experimentally demonstrated the inhibitory abilities of the selected compounds in vitro, as well as the anti-tumor efficacy in a tumor-bearing mice model.

## 2. Results

### 2.1. Strategic Overview of Drug Repurposing

For the first stage of drug repurposing, we used the crystal structure of NICD–MAML–RBPJ–DNA complex (PDB ID: 6PY8) to perform the RBPJ-based virtual screening. Crystal structure has revealed that RBPJ protein contains three domains: BTD (β-trefoil domain), NTD (the N-terminal domain), and CTD (the C-terminal domain) (Supplementary Material Figure S2). In this complex, the RBPJκ–associated module (RAM) domain of Notch intracellular domain (NICD) binds the BTD of RBPJ, showing that five residues (Val223, Phe221, Met243, Pro246, and Gln293) in BTD are important for the binding (Supplementary Material Table S1). Also, the Ankyrin repeat (ANK) domain of NICD binds the CTD of RBPJ, which identifies seven key residues of BTD (Gly350, Gln347, Leu348, Glu358, Glu385, Cys383, and Gly384). One transcriptional coactivator, MAML, interacts with the CTD-ANK interface and the NTD of RBPJ, revealing the key residues of RBPJ (Arg382, Arg378, Tyr381, Asn367, Arg369, Asn417, Lys130, Asp138, Phe88, Gly134, Ser136, Met98, and Cys86) (Supplementary Material Table S2). In light of the interaction between DNA and RBPJ, the crucial residues of RBPJ to maintain the interaction includes Lys44, Tyr46, Lys50, Arg51, Phe52, Ser151, Ser154, Gln158, Arg178, Arg180, Ser181, Gln182, and Lys271.

**Figure 1:**
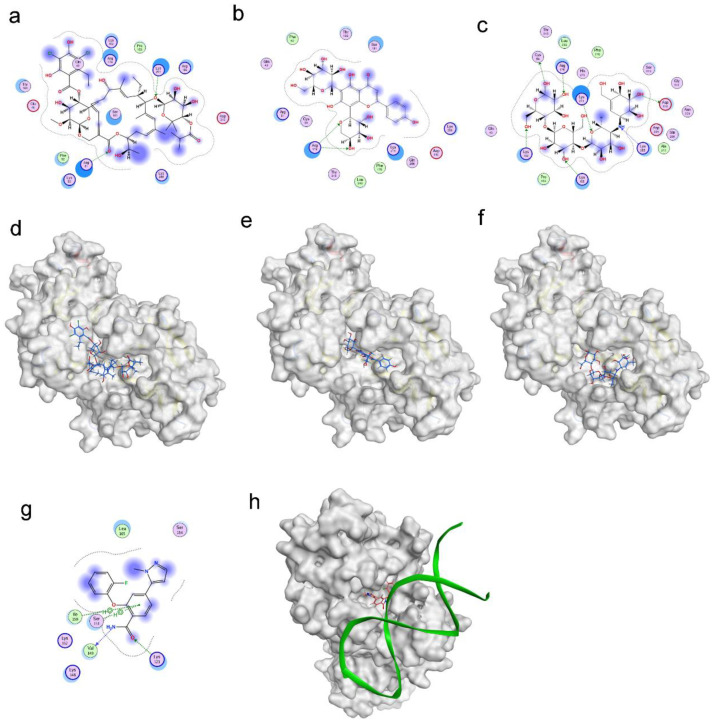
Molecular docking revealed binding modes of three hits and positive control with RBPJ protein by using MOE. Two-dimensional and three-dimensional docking poses of fidaxomicin (**a**,**d**), schaftoside (**b**,**e**), acarbose (**c**,**f**), and positive control RIN1 (**g**,**h**) at active sites of RBPJ were represented, respectively, showing the shape complementarity and the H-bonding with key contributing amino acids of RBPJ.

The virtual screening for RBPJ inhibitor was conducted using the docking module of MOE software(2020.09). We collected 10,527 compounds from various sources, including TCMSP, PubChem, DrugBank, and TOPSCIENCE Bioactive compound. Each compound in this collection was first subjected to blind docking, in which each one globally docked onto the entire surface of the RBPJ protein without any prior knowledge (PDB ID: 6PY8). 21 hits were selected from blind docking, and they exhibited docking scores less than −8 kcal/mol. These 21 molecules were further site-specifically docked into RBPJ based on the identified key amino acid residues of RBPJ. Three compounds, including fidaxomicin, schaftoside and acarbose, were identified to be the strongest RBPJ binder as they not only occupied the binding site strictly but also formed hydrogen bonds with multiple key RBPJ residues within the binding site (Figure 1). A positive control, RIN1 [19], was employed to further confirm the putative binding site of the RBPJ protein by molecule docking. The chemical structures of three hits and RIN1 were shown in Supplementary Material Figure S1. RIN1 has previously been shown to be capable of interrupting the formation of RBPJ-dependent transcriptional complex. The DNA-binding site of the RBPJ protein, as shown in Figure 1g,h, was also favored by RIN1, although only one molecule of RIN1 could not repel the DNA molecule from its binding site (Figure 1h). On the basis of the result of RIN1 docking investigation, it suggested that the selected three hits could potentially inhibit RBPJ-dependent transcription.

### 2.2. Evaluation of the Inhibitory Abilities of Three Selected Compounds

**Figure 2:**
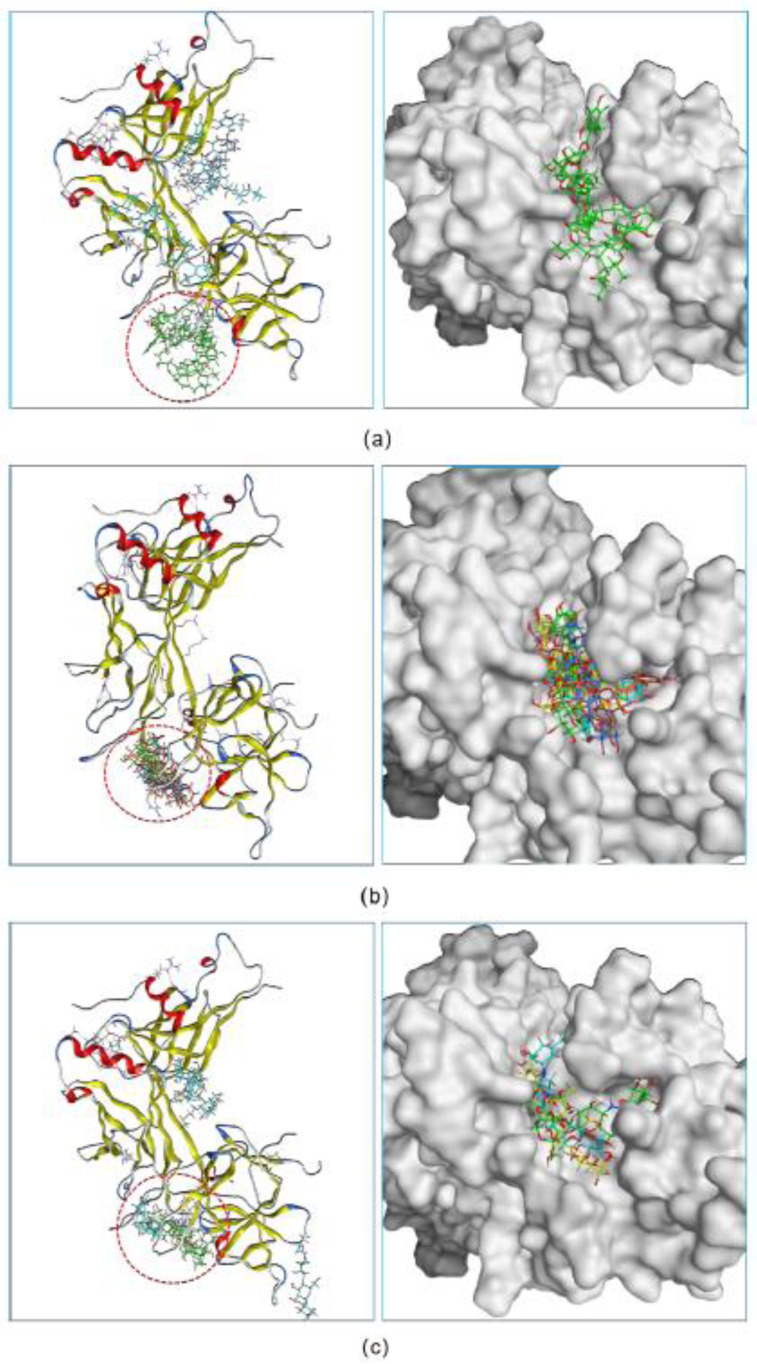
The inhibitory abilities of three hits were investigated in a docking experiment. Three fidaxomicin molecules (**a**), or four schaftoside molecules (**b**), or three acarbose molecules (**c**) could strictly occupy the active site of RBPJ that is indicated by a red dashed circle.

Following the screening results, we further investigated the potency of three hits to inhibit the formation of the NICD–MAML–RBPJ–DNA transcriptional complex. For each hit, docking was used to determine the maximum number of compound molecules that could simultaneously bind to the active site of RBPJ. The docking procedure began with the docking of a single molecule onto the RBPJ, resulting in the top scored pose in which the molecule bound to the active site. Then, using this resultant pose as the starting structure for the secondary round of docking, another molecule was docked onto this complex, yielding the top scored pose containing two molecules docked to the active site. This docking step was repeated several times until the *n*th input molecule was unable to bind to the RBPJ active site that was previously occupied by *n*-1 molecules. It is suggested that the more molecules bound to the active site, the more effective the compound would be at inhibiting the function of RBPJ. As shown in Figure 2a, three fidaxomicin molecules were positioned in such a way that all of them could simultaneously fit into the active site; this active site could hold up to four schaftoside (Figure 2b) or three acarbose molecules (Figure 2c).

**Figure 3:**
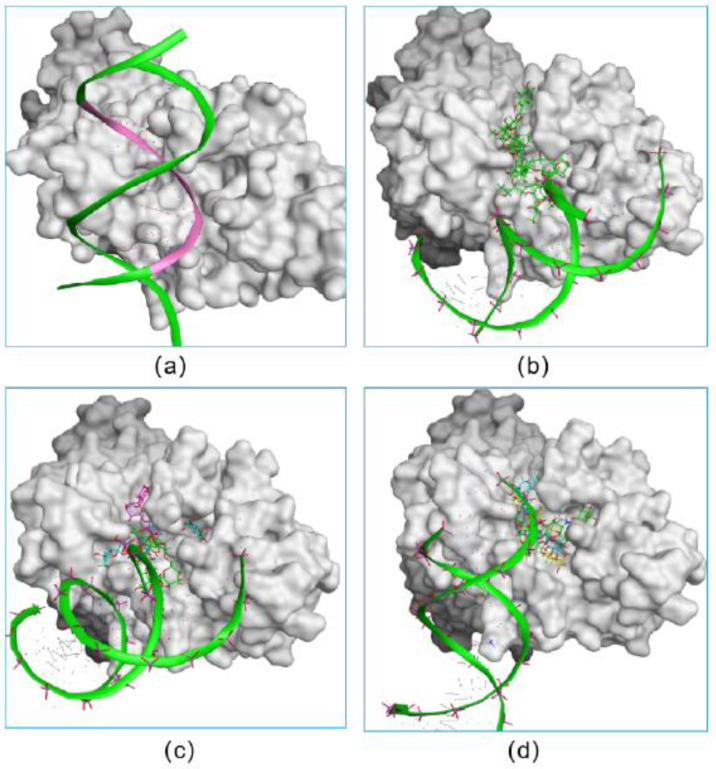
The verification on the abilities of small inhibitors to inhibit the formation of the NICD–MAML–RBPJ–DNA complex. The original DNA bound complex is shown (**a**). The occupancy of three fidaxomicin (**b**), four schaftoside (**c**) or three acarbose (**d**) molecules in the active site would inhibit the binding of DNA, indicating the DNA-competitive ability of the three hits.

In addition, we investigated the DNA-competitive ability of each hit, which could further validate the potential of selected compounds as an RBPJ-specific inhibitor. To be specific, the obtained pose consisting of RBPJ and compounds was inputted as the docking receptor for the docking with the DNA molecule. As shown in Figure 3, the DNA molecule was pushed to dock onto other sites of RBPJ when various molecules of each hit occupied the active site, which confirmed the three hits to be DNA competitors and their ability to inhibit the formation of the NICD–MAML–RBPJ–DNA complex.

### 2.3. Molecular Dynamic Simulations

**Figure 4:**
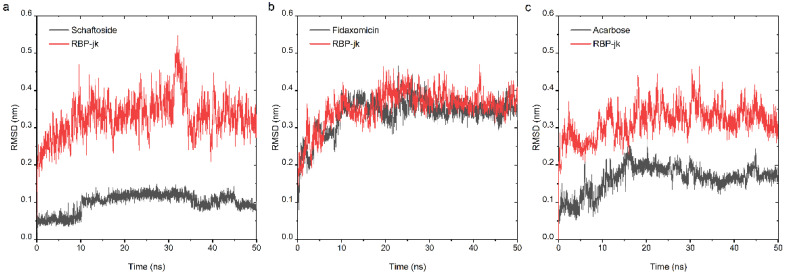
Analysis of RMSD trajectories for the complex of the RBPJ protein and selected hit throughout 50 ns all-atom MD simulation. RMSD plots for schaftoside (**a**), fidaxomicin (**b**), acarbose (**c**) in complex with RBPJ were illustrated, respectively. Each compound was shown in black and RBPJ protein shown in red. RMSD, root-mean-square deviation.

At the second stage of drug repurposing, we further performed an all-atom molecular dynamic (MD) simulation to investigate the dynamic behavior of both selected hits and RBPJ and to assess their binding stability. The obtained poses consisting of each hit and RBPJ were subjected to 50 ns all-atom MD simulation. The stability profile of three hits in complex with RBPJ was examined by using their respective root mean square deviation (RMSD) values throughout all the simulation runs. As RMSD provides the degree of conformational variability of a protein, a ligand, or a ligand–protein complex, a high RMSD value for one ligand would indicate its incompatibility with the active site of the protein, as well as insufficient stability of the ligand–receptor complex across MD simulation time-frames. RMSD values for the Cα backbone of RBPJ and each compound were calculated and are shown in Figure 4. For the schaftoside–RBPJ complex, both RMSD curves of schaftoside and RBPJ exhibited relatively stable fluctuations with the fluctuation amplitude being less than 0.1 nm, suggesting that the MD simulations reached equilibrium after 10 ns and schaftoside bound tightly to the active site of RBPJ. Similarly, fidaxomicin and acarbose formed stable complexes with RBPJ because both systems were well equilibrated after 30 ns and yielded relatively steady RMSD values.

The MM-PBSA method was used to calculate the binding free energies of ligand-protein complex and to rank the binding affinity of three compounds [27,28,29]. The binding free energy in this method is decomposed into different components, including intermolecular van der Waals (ΔE_vdW_), electrostatic interactions (ΔE_elec_), non-polar solvation energy (ΔG_np_), polar solvation free energy (ΔG_pol_) and the configurational entropy (-TΔS). The components of the binding free energies of three complexes were listed in Table 1. The van der Waals (ΔE_vdW_), electrostatic interactions (ΔE_elec_) and non-polar solvation energy (ΔG_np_) drove the complexation of each hit to RBPJ, while polar solvation free energy (ΔGpol) and the configurational entropy (-TΔS) worked against the binding. As shown, the calculated binding free energy of fidaxomicin was the lowest among the three, indicating that fidaxomicin could form the most stable complex with RBPJ. Schaftoside showed the second-ranked favorable binding energy. However, acarbose exhibited a positive binding free energy, which indicated an energetically unfavorable interaction between acarbose and the active site of RBPJ. Taken together, this finding further suggested fidaxomicin to be the strongest binder among the three.

### 2.4. In Vitro Cytotoxicity Analysis and Intracellular Trafficking

**Figure 5:**
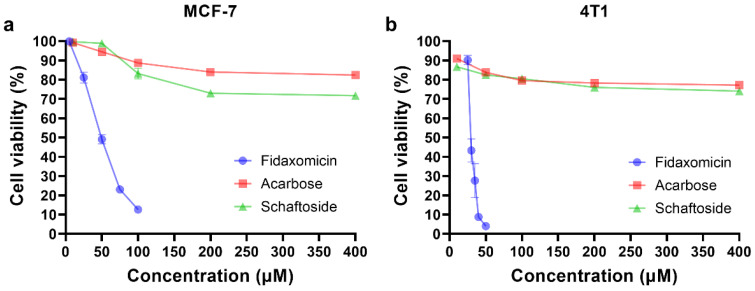
In vitro cytotoxicity analysis. (**a**) Cytotoxicity test in human breast cancer MCF-7 cells. (**b**) Cytotoxicity test in mouse breast cancer 4T1 cells. The cells were incubated with various concentrations of fidaxomicin, acarbose, or schaftoside for 48 h, and the viability of cells was examined by CCK-8 assay (*n* = 5 samples per group).

Encouraged by the results of docking and molecular dynamic simulations, the inhibitory properties of three compounds were determined in different cell lines. As shown in Figure 5, fidaxomicin was active at micromolar concentrations to inhibit human breast cancer cell MCF-7 and mouse breast cancer cell 4T1, with IC_50_ values of 53.4 and 32.3 μM, respectively. In comparison, schaftoside and acarbose did not exhibit significant anti-tumor effects against MCF-7 and 4T1 across the concentration range that was investigated (Figure 5). Collectively, the anti-tumor efficacy of fidaxomicin was evidently superior to that of other two screened compounds in vitro.

**Figure 6:**
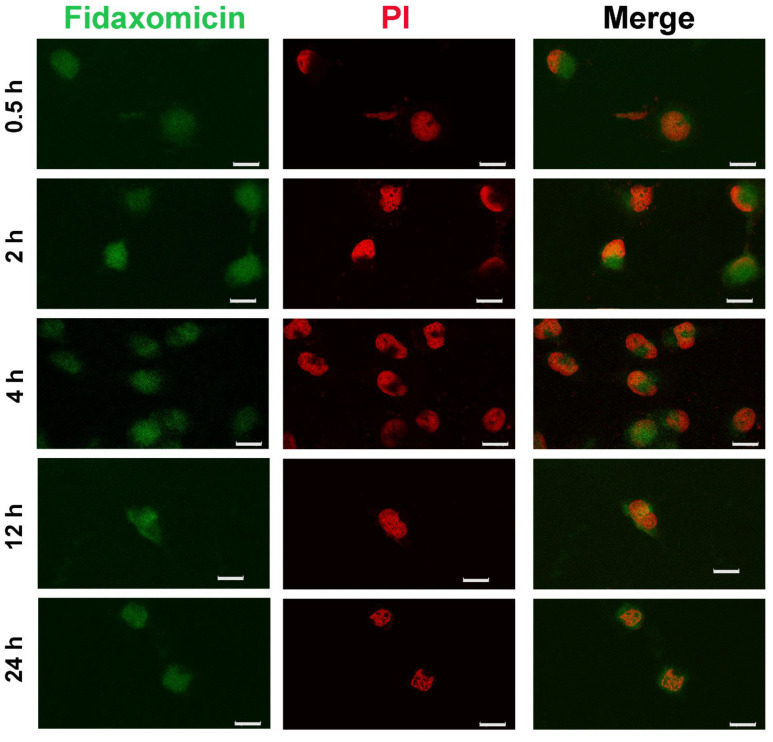
Cellular internalization of fidaxomicin in 4T1 cells. The confocal fluorescence images of 4T1 cells were obtained after incubation with fidaxomicin for 0.5, 2, 4, 12, and 24 h. Scale bar: 20 μm.

Next, to ensure that the anti-tumor ability of fidaxomicin resulted from inhibiting the formation of the transcriptional complex, effective internalization by tumor cells and intracellular nucleus targeting were of great importance. Here, CLSM was used to investigate the subcellular localization of fidaxomicin in 4T1 cells (Figure 6). The relatively weak fluorescence signal (green) of fidaxomicin was observed in the nuclei (red) of 4T1 cells after 0.5 h incubation, and enhanced fluorescence of fidaxomicin could be detected after 2 h and 4 h incubation, indicating that the fluorescence intensity of fidaxomicin in cell nuclei exhibited a time-dependent increase. These results confirmed that fidaxomicin was effectively taken up by 4T1 cells and then distributed into the cell nuclei. Furthermore, due to the nuclear location of the RBPJ protein, the intracellular trafficking behavior of fidaxomicin implied that this compound might inhibit the formation of the RBPJ complex, hence blocking the Notch signal pathway and eventually exerting the potential anti-tumor ability.

### 2.5. In Vitro Evaluation of RBPJ-Specific Inhibition

**Figure 7:**
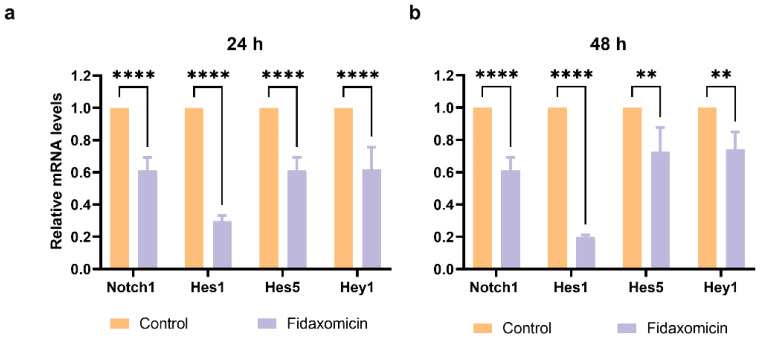
The mRNA levels of Notch target genes in 4T1 cells after incubation with fidaxomicin for a variety of times. (**a**) The results of a 24 h incubation with fidaxomicin; (**b**) the results of a 48 h incubation with fidaxomicin. qRT-PCR analysis revealed that the incubation of fidaxomicin significantly reduced the mRNA levels of Notch1, as well as Notch target genes Hes1, Hes5, and Hey1. Data shown represent the mean ± SD of triplicate experiments (** *p* < 0.01, and **** *p* < 0.0001 vs. control group).

The ability of fidaxomicin to disrupt the interaction between NOTCH, RBPJ, and DNA was further confirmed by quantitative real-time PCR (qRT-PCR) analysis. The gene encoding Notch receptor Notch1 and Notch downstream target genes, including Hes1, Hes5, and Hey1 in 4T1 cells after incubation with fidaxomicin for 24 h and 48 h were analyzed (Figure 7). As a result, the 24-h treatment of fidaxomicin significantly repressed the amounts of Notch1, Hes1, Hes5, and Hey1 mRNAs. We observed that the expression of Hes1 was the most significantly repressed, and the repression of Notch1 and Hes1 responded to fidaxomicin treatment in a time-dependent manner. The decreases of Hes5 and Hey1 mRNA were less prominent than those of the other two genes, and they did not show a further decrease after 48 h incubation of fidaxomicin.

**Figure 8:**
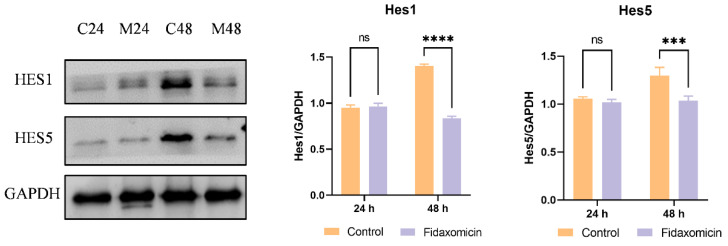
Western blot analysis of protein levels of Notch target molecules in 4T1 cells. C24 and C48 denote the protein levels in untreated 4T1 cells after culture for 24 h and 48 h, respectively. M24 and M48 indicate the protein levels in cells after fidaxomicin treatment for 24 h and 48 h, respectively. After incubation, whole-cell extracts were subjected to immunoblot analysis and incubated with antibodies to Hes1, Hes5, and GAPDH as a control. Data shown represent the mean ± SD of triplicate experiments (*** *p* < 0.001, and **** *p* < 0.0001 vs. control group, ns: no significance).

Additionally, the protein levels of Notch downstream targets Hes1 and Hes5 were analyzed by western blotting. Despite the decrease in mRNA levels, fidaxomicin did not appear to affect the expression of Hes1 and Hes5 proteins in 4T1 cells during the first 24-h incubation, as shown in Figure 8. However, following 48-h incubation with fidaxomicin, the protein levels of Hes1 and Hes5 were significantly reduced, which was consistent with the mRNA alterations in both genes after the same treatment. Combined with in vitro cytotoxicity and qRT-PCR results, these findings implied that fidaxomicin might effectively block the formation of the RBPJ-dependent transcriptional complex, leading to the inhibitory ability on Notch signaling and resulting in anti-tumor activity.

### 2.6. Antitumor Efficacy

**Figure 9:**
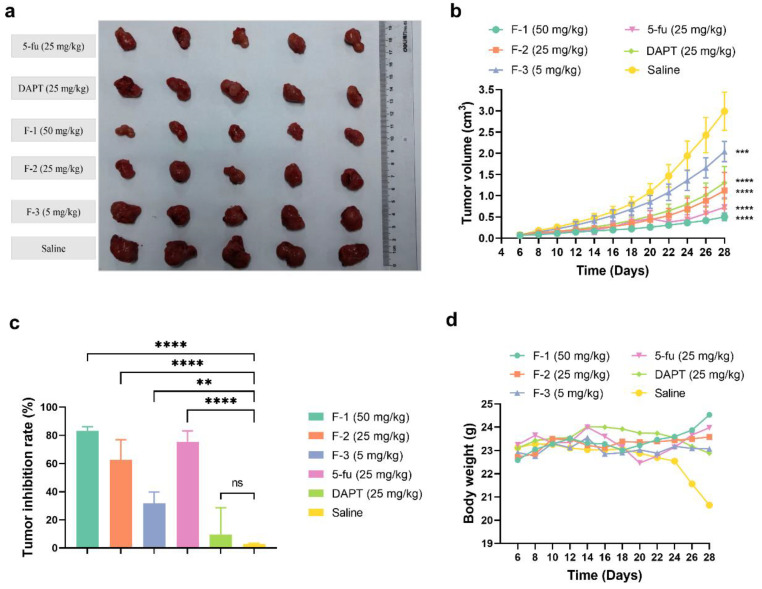
The in vivo antitumor effects of fidaxomicin in 4T1 tumor-bearing mice. (**a**) The photograph of tumors excised from sacrificed mice. (**b**) Tumor growth curve, (**c**) tumor inhibition rate, and (**d**) body weight profile were demonstrated after different treatments. Data shown represent the mean ± SD (*n* = 5, ** *p* < 0.01, *** *p* < 0.001, and **** *p* < 0.0001 vs. control group, ns: no significance).

Based on the in vitro cytotoxicity and the responses of inhibiting the Notch signaling, the antitumor efficacy of fidaxomicin in 4T1-tumor-bearing mice was then evaluated. When the average tumor volume reached about 100 mm^3^, mice were treated with different groups of drugs by intratumoral administration every other day for three weeks, including 5, 25, and 50 mg/kg fidaxomicin, 25 mg/kg DAPT, 25 mg/kg 5-fluorouracil, and saline as a negative control. As shown in Figure 9a,b, the tumor volume of the saline group increased rapidly and reached 3000 mm^3^ on the 28th day after the implantation.

The growth curves of tumors revealed that the order of the inhibitory ability on tumor growth was fidaxomicin (50 mg/kg) > 5-fluorouracil (25 mg/kg) > fidaxomicin (25 mg/kg) > DAPT (25 mg/kg) > fidaxomicin (5 mg/kg) (Figure 9b). The low dose of fidaxomicin (5 mg/kg) exhibited a moderate tumor inhibition compared with the saline group. When the dose of fidaxomicin was further increased, stronger inhibitions were observed. As a result, fidaxomicin demonstrated potent anti-cancer activity, yielding 62.56% and 83.19% inhibitory efficacies on the growth of tumors at 25 and 25 mg/kg, respectively (Figure 9c). In comparison, DAPT, a well-known Notch inhibitor that inhibits the activity of gamma-secretase, exhibited an inhibition rate of 56.42% at a concentration of 25 mg/kg, whereas 5-fluorouracil, another extensively used chemotherapeutic drug, showed a better inhibition rate of 75.66% at the same dosage. The average tumor weight of mice treated with fidaxomicin was significantly lower than that of mice treated with DAPT or 5-fluorouracil at a dose of 25 mg/kg, indicating the greatest anti-tumor ability among the three treatment groups. In addition, there was no noticeable body weight change or obvious abnormalities in the three fidaxomicin groups, indicating that fidaxomicin was tolerable up to 50 mg/kg when administered 12 times (Figure 9d).

**Figure 10:**
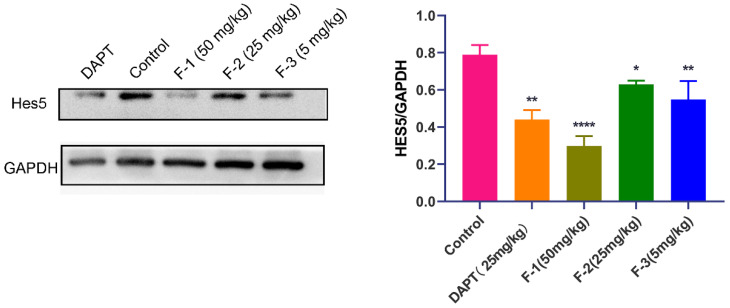
Relationship between in vivo anti-tumor activity of fidaxomicin and the inhibition in Notch signaling in 4T1 tumors. Western blot analysis of protein levels of Notch targets Hes5 in 4T1 tumors was performed after administration with saline, DAPT (25 mg/kg), fidaxomicin (50 mg/kg), fidaxomicin (25 mg/kg), and fidaxomicin (5 mg/kg). Data shown represent the mean ± SD of triplicate experiments (* *p* < 0.05, ** *p* < 0.01, and **** *p* < 0.0001 vs. control group).

We further verified the relationship between the in vivo anti-tumor efficacy of fidaxomicin and the inhibition of Notch signaling in 4T1 tumor-bearing mice. As shown in Figure 10, the protein levels of Hes5 were significantly reduced in the tumor grafts of mice treated with fidaxomicin in a dose-dependent manner in comparison with the saline control groups. The high dose of fidaxomicin could reduce the expression of Hes5 by 65.7%, implying that the anti-tumor activity of fidaxomicin was correlated with its possible inhibitory ability in Notch signaling. The positive control DAPT also reduced the expression of Hes5 at a dose of 25 mg/kg, showing that DAPT exerted its ability to block Notch signaling through the γ-secretase inhibitor.

## 3. Discussion

In this study, we demonstrated that fidaxomicin was identified as a potential small molecule inhibitor of RBPJ, eventually inhibiting Notch signaling in breast cancer cells. Since the Notch signal pathway regulates the differentiation of breast epithelial cells during normal development, aberrant Notch signaling, as seen by increased NICD release and target gene overexpression, is positively correlated with poor patient prognosis, such as aggressive, metastatic triple negative breast cancer (TNBC) and therapy resistance [30,31,32]. In light of these findings, the inhibition of overactive Notch signaling would be facilitated in the treatment of breast cancer. Regarding the function of RBPJ, the RBPJ–NICD–DNA complex induces transcription of Notch target genes including various Hairy/Enhancer of Split-related genes (Hes1, Hes5, and Hey1) [18,33]. In recent years, the involvement of these Hes/Hey canonical Notch target genes in breast cancer initiation and progression has been recognized. For example, Hes1, a basic helix–loop–helix (bHLH) transcriptional repressor, is an important direct RBPJ-dependent Notch target gene. The proliferation and invasion of TNBC cells were boosted by the overexpression of Hes1, while on the contrary, this effect was substantially abolished by silencing Hes1 gene expression [34]. Therefore, compared with GSIs, suppressing RBPJ-dependent gene transcription would confer advantages including direct mediation on Notch signaling as well as reduced side effects.

The drug repurposing strategy in this study was a combination of molecular docking and dynamic simulation. Molecular docking is a well-established method for screening potential compounds based on their complementarity with the target’s binding site. We started by building a database of FDA-approved drugs, synthetic compounds, and natural extracts to be used in the initial screening study. The fact that RBPJ interacts with multiple components, including NICD, certain coactivators, and DNA, led us to first investigate the binding mode between each component and the RBPJ, identifying the possible binding sites and the key residues in these binding sites. After evaluating the docking scores of all compounds, 21 compounds with high docking scores were selected to dock onto the binding sites of RBPJ. Interestingly, we found that three hits preferred the sites where large molecule DNA binds rather than other coactivators and NICD, which suggested that the DNA binding site could be a possible target for the design of RBPJ inhibitors. To further prove that the three hits could effectively compete with DNA for RBPJ binding sites, we used docking to demonstrate how more than two molecules could fully occupy the DNA-binding site, causing DNA to weakly interact with the remaining sites. Based on these docking results, we were able to identify novel roles for the three hits as potential RBPJ-specific inhibitors.

Among the three hits, fidaxomicin is a first-in-class macrocyclic antibiotic for the treatment of *Clostridium difficile* infection. Regarding its mechanism of action, fidaxomicin primarily inhibits bacterial RNA polymerase at an early step in the transcription initiation stage. Specifically, fidaxomicin binds to the σ subunit of bacterial RNA polymerase, preventing the initial separation of DNA strands [35,36]. Other than the treatment of Gram-positive bacteria, fidaxomicin has not been found to be effective in any other application. In this study, we identified a novel role of fidaxomicin as a potential anti-tumor candidate. We further investigated the binding site for fidaxomicin by superimposing the complex docked with fidaxomicin over the RBPJ-dependent transcriptional complex. Interestingly, we discovered that the pocket of RBPJ that was occupied by fidaxomicin was open for DNA binding, whereas fidaxomicin had no effect on the interaction between RBPJ and NICD or other coactivators. As a result, the RBPJ transcriptional complex would not be formed, eventually inhibiting the expression of Notch downstream target genes. Another hit, acarbose, is an α-glucosidase inhibitor and is used to manage glycemic control in patients with type 2 diabetes mellitus [37,38]. As an oligosaccharide, acarbose inhibits the activities of several enzymes responsible for the hydrolysis of complex carbohydrates in the small intestines. The third hit schaftoside was a flavonoid that has been found in Chinese medicinal herbs. Previous studies have revealed that schaftoside has a variety of pharmacological activities, including anti-inflammatory [39,40], anti-melanogenic [41], anti-stress, and antioxidant capacities [39,42,43]. In light of the blind docking results, acarbose and schaftoside preferred to dock onto the DNA-binding pocket of RBPJ, which was also observed in top-ranked poses of a fidaxomicin–RBPJ complex. These docking poses implied that the two other hits would inhibit the formation of the RBPJ-related transcriptional complex in a similar manner to fidaxomicin.

Further docking experiments revealed that the highest number of molecules that could occupy the active site of RBPJ was three for fidaxomicin, four for schaftoside, and three for acarbose. Next, to investigate whether the compounds were DNA competitors, we studied the behavior of the selected compounds when DNA were docked to the pose of the RBPJ-selected compound complex. This result indicated that any of the three selected compounds could strictly occupy the DNA-binding site of RBPJ, thus causing DNA to interact weakly with another site of RBPJ. Taken together, our virtual screening and docking experiments revealed that the three identified compounds were potential RBPJ-specific inhibitors, interacting with the DNA-binding site of RBPJ and eventually exerting inhibitory ability on the expression of Notch target genes.

At the second stage of our drug repurposing strategy, dynamic simulation was employed to further refine the docking results by analyzing the binding properties of each identified compound, such as the binding free energy. Here we selected the molecular mechanism/Poisson–Boltzmann surface area (MM-PBSA) approach to obtain the binding free energy of each hit-RBPJ complex. However, although the computational cost of MM-PBSA is far lower than that of other approaches, which is a significant advantage, this method suffers from a higher number of computational errors and lower accuracy compared with other methods [44,45,46]. As a result, we used this approach to rank the binding free energies of the three hits in order to determine which one might be the most promising RBPJ inhibitor. The MM–PBSA results revealed that fidaxomicin performed the best, showing the lowest binding free energy of −76.555 kJ/mol. The binding free energy of schaftoside was −43.72 kJ/mol, which was ranked second. Surprisingly, acarbose revealed a positive binding free energy, which was not suitable for the interaction between acarbose and RBPJ. Because acarbose is a water-soluble oligosaccharide, its physicochemical properties might make it difficult to access the DNA-binding site of RBPJ, resulting in an unfavorable binding free energy. In addition, docking scores are assessed mainly based on the shape complementarity between candidate and target receptor, rather than the binding behavior of the candidate in a real situation with a given salt concentration, so the discrepancy between the docking experiment and dynamic simulation might be explained.

To validate the results of our drug repurposing, the anti-tumor activities of three hits were determined by cellular viability assay. Among them, fidaxomicin exhibited a potent anti-tumor effect against two breast cancer cell lines, with IC_50_ values in a micromolar range. When incubated with either the human breast cancer cell line MCF-7 or mouse breast cancer cell line 4T1 for 48 h, schaftoside was found to have negligible toxicity for both cell lines at concentrations up to 400 μM. Another hit, acarbose, showed poor anti-tumor effect against two cell lines, similar to that of schaftoside. Among the three hits, only fidaxomicin had the lowest IC50 values, i.e., 53.4 μM for MCF-7 and 32.3 μM for 4T1. Regarding the poor anti-tumor performance of schaftoside and acarbose, both compounds might not have good permeability that largely depends on their lipophilicity, molecular weight, and polarity [47]. For example, acarbose is a complex oligosaccharide and is soluble in aqueous solution [48], so it might not be able to penetrate across cellular membranes and subsequent nucleus membranes. Therefore, although the three hits could theoretically inhibit the formation of the RBPJ-dependent transcriptional complex, schaftoside and acarbose might not passively diffuse into the cell nucleus; on the contrary, fidaxomicin was proved to be distributed in the cell nucleus after incubation for 2 h by using CLSM, which met the requirement for the RBPJ-specific interaction. Further studies would be conducted to determine the cause of the poor performance of schaftoside and acarbose, and the application of appropriate delivery systems could help these compounds distribute to the nucleus, where they could then exert inhibitory effects on Notch signaling.

To establish whether fidaxomicin inhibits RBPJ in cells, the expressions of Notch target genes at the level of transcription and translation were assessed after compound treatment by using qRT-PCR and western blotting, respectively. Figure 7 summarized the results of qRT-PCR when 4T1 cells were incubated with fidaxomicin for either 24 h or 48 h. The reduced levels of Notch target genes, including Hes1, Hes5, and Hey1, were detected after fidaxomicin treatment, indicating that the RBPJ-mediated transcription was inhibited in cells. The protein levels of Notch targets Hes1 and Hes5 were significantly reduced after 48 h incubation of fidaxomicin, further confirming the fidaxomicin-induced inhibition of Notch signaling. Notably, the expressions of the two proteins were not significantly altered during the first 24 h incubation of fidaxomicin, which might be due to their functions. The Hes1 protein contains both DNA-binding and protein–protein interaction domains responsible for its role as a transcriptional regulator, which negatively regulates its own transcription [49,50]. Therefore, the already-existing Hes1 protein would still exert its activity and regulate the expression during the first 24-h incubation, resulting in a marginally declined protein level; when fidaxomicin exposure was extended to 24 h to 48 h, a significantly lower level of the Hes1 protein was observed. Hes5 is another transcriptional regulator, and the treatment with fidaxomicin caused a similar change in the level of the Hes5 protein to that observed with Hes1 level. This result could be explained by the fact that Hes5 has been reported to negatively control its transcription in neurogenesis and hepatocarcinogenesis [51,52,53,54]. Collectively, these results demonstrated that fidaxomicin might act as a potential RBPJ-specific inhibitor and effectively block Notch signaling.

Fidaxomicin’s potential to inhibit the growth of 4T1 tumors was further demonstrated in a 22-day in vivo anticancer experiment. At the highest dose of 50 mg/kg, this compound was able to reduce tumor volume by 83.19%, and it also had a modest anti-tumor impact at a dose of 5 mg/kg, resulting in a decrease in tumor volume of 31.77%. To assess the anti-tumor potential, we used positive controls including a commonly used GSI (DAPT) and an established chemotherapeutic agent (5-fluorouracil). As a result, at a dose of 25 mg/kg, fidaxomicin had a better anti-tumor effect (62.56%) than DAPT (56.42%). On the other hand, fidaxomicin was not as effective against 4T1 tumor as 5-fluorouracil, which showed a reduction in tumor volume of 75.66% when given at the same dose.

The mechanism of action of fidaxomicin against tumors was elucidated by measuring Hes5 protein levels in tumor tissues. Since the levels of the Hes5 protein decreased more slowly than that of Hes1 in in vitro cellular studies, the changes in the Hes5 levels may better reflect the inhibitory effect of fidaxomicin on RBPJ-mediated transcription. In accordance with in vitro results, the levels of Hes5 proteins in tumor tissues were considerably decreased after mice received various doses of fidaxomicin, suggesting a possible RBPJ-dependent inhibitory mechanism of action. These findings matched the in vitro cellular assay results, indicating that the in vitro cytotoxicity was well translated into in vivo activity.

One limitation of our study is that we did not have the appropriate cell- or animal-based models to regulate the activity of the RBPJ protein at the time of our study, which would have allowed us to directly examine the inhibitory ability of selected hits on RBPJ function. Based on our findings, fidaxomicin has been proposed as a potential RBPJ-dependent inhibitor. However, further work is required to verify the binding mode of fidaxomicin with RBPJ as well as the precise mechanism of action. Another limitation of our study is that we did not investigate the phenotypes that were responsible for Notch signaling, such as the development of splenic marginal zone B (MZB) cells, thymic T cell development, generation of Esam^+^ dendritic cells, sprouting of endothelial cells, and induction of goblet cell differentiation in the small intestine. CB-103 [20], a small molecule Notch inhibitor, was previously shown to reduce these phenotypes when administered orally. Although fidaxomicin in this study was injected intratumorally in this study, which might not have resulted in these phenotypes, we plan to leverage the potential systemic delivery of fidaxomicin and address this limitation in the future research.

In contrast to the widely used GSIs, our results suggested a promising future for the application of fidaxomicin. In spite of the fact that fidaxomicin is not as potent as currently available drugs, it represents a promising starting point for the development of a potential RBPJ-specific inhibitor, which might be further improved by using in silico design cycles.

## 4. Materials and Methods

### 4.1. Structure Preparation of the Screening Library

We built a virtual chemical library, a collection of 10,527 pharmacologically active compounds, by integrating four publicly available databases: (1) the DrugBank database (https://go.drugbank.com/, accessed on 1 June 2020); (2) the PubMed database (https://pubchem.ncbi.nlm.nih.go, accessed on 1 June 2020); (3) Traditional Chinese Medicine Systems Pharmacology (TCMSP) database (https://tcmspw.com/tcmsp.php, accessed on 1 June 2020) [55]; and (4) TOPSCIENCE Bioactive compound library (https://www.tsbiochem.com/ accessed on 1 June 2020). This library covered the major classes of compound candidates, including FDA-approved drugs, investigational new drugs, preclinical compounds, drug-like chemicals, and natural products with immunomodulating effects. The 3D structures of all these compounds were prepared, and in turn minimized using default parameters through Molecular Operating Environment (MOE 2019) software in terms of geometry and energy.

The three-dimensional crystal structure of the RBPJ–NOTCH1–NRARP ternary complex was downloaded from the Protein Data Bank (PDB) (PDB ID: 6PY8) and used as the target for the screening within this study. Next, we analyzed potential sites for the binding of ligand and DNA molecules to the RBPJ protein and restriction sets for rendering partial molecular surfaces, showing possible docking sites on the RBPJ protein. In turn, we removed all solvent molecules, ligands (NOTCH1 and NRARP) and DNA from the complex and refined the RBPJ protein by adding hydrogen atoms, filling in missing side chains and loops, and assigning hydrogen bonds through the Protein-Structure Preparation module of MOE 2019. The structure of RBPJ was then subjected to energy minimization using MOE with default parameters as mentioned above.

### 4.2. Docking Procedure

#### 4.2.1. Blind Docking Studies to Screen Candidates

Molecular docking between RBPJ protein and each compound in the virtual screening library was performed using the docking module of MOE. A three-stage docking procedure was conducted to identify a potential Notch inhibitor. During the first stage, blind docking was employed to quickly dock the entire target surface with multiple binding sites.

The docking procedure at the first stage was applied for screening the significant candidates from the library. Briefly, selections of “Receptor” and “Ligand” in the MOE Dock module were assigned to corresponding structures, where “Site” was assigned to “All atom”, which meant that each candidate was globally docked onto the RBPJ structure without any specified binding site, namely, blind docking. For each test compound, 100 different poses were generated and scored according to the default London dG scoring function, and in turn the best 10 poses were selected. During the screening, values of additional parameters remained unchanged. To select the potential candidate, the retention value was set as the binding energy less than −8 kcal/mol, and the location of the binding site was nearby the active binding sites.

Clustering of the generated poses was conducted to achieve the largest cluster, and in turn, each compound in the best possible cluster was evaluated on the basis of docking scores less than −8 kcal/mol, binding mode within the RBPJ active site, and molecular interactions with the key residues of the RBPJ. After completion of this stage of the docking process, 21 hits were selected.

#### 4.2.2. Restricted Active Site Docking

The docking study at the second stage was a more directed screening which was carried out with the 21 obtained hits. Unlike the previous “blind docking”, the docking search at this stage focused on the key residues of RBPJ that were involved in the interactions between the RBPJ protein and NICD or DNA. In addition to the docking score that should be less than −8 kcal/mol, the tested compound that could interact with more than three key residues of RBPJ and occupy a large binding space was retained for the next stage of the investigation. According to these screening criteria, three compounds were finally selected.

#### 4.2.3. DNA-Competitive Docking Studies

To further verify the potent Notch-inhibiting ability of the obtained hits, a third-stage advanced docking study was performed. In this docking search, we examined the highest possible number of each tested compound that could simultaneously bind to the same active site of RBPJ. The docking parameters were as same as in blind docking that used in the first stage, but the receptor structure was different. In detail, one molecule, in the first blind docking cycle, bound to the target binding site and formed a complex, the best conformation of which was chosen as the receptor structure for the next blind docking cycle; as a result, a new complex containing an increasing number of tested compounds was generated in each blind docking cycle. Until an input compound could not stably bind to the active site of the complex from the previous cycle, the docking study finally came to an end. This docking study resulted in a top-ranked pose of the complex consisting of one RBPJ and several compound molecules. Clearly, the higher the number of compound molecules that occupied the active site of RBPJ, the stronger the ability of this compound to inhibit the Notch activity.

Next, to investigate the capacity of the selected compound to inhibit the formation of the RBPJ-dependent transcriptional complex, we carried out the DNA-competitive docking experiment. Briefly, the obtained pose of the complex, which contained one RBPJ and the maximum number of selected compound molecules, was used as receptor structure, and the DNA molecule was inputted in this round to dock onto the complex. Then, we studied the position of the DNA in the obtained pose, showing whether the selected compound could effectively occupy the DNA-binding site of RBPJ.

After completion of the three-stage docking study, the identified compounds were further evaluated by molecular dynamic simulations.

### 4.3. Molecular Dynamics (MD) Simulations

#### 4.3.1. MD Simulation Procedure

The most promising compounds, fidaxomicin, schaftoside, and acarbose, in complex with the RBPJ protein were further evaluated using all-atom MD simulations, which were performed with the GROMACS software package (Version 2021.1, Royal Institute of Technology and Uppsala University, Sweden) using the AMBER99SB-ILDN forcefield parameter set. The topologies of three compounds in the GROMACS format were generated by the ACPYPE tool [56], where General Amber Force Field (GAFF) was used. For the MD simulation, the amber99SB-ILDN force field was used to parameterize the RBPJ protein. The complex consisting of the ligand and RBPJ protein was placed in a dodecahedron box, which was solvated with the TIP3P water model with a 10 angstrom padding region. Na^+^ ions were added to neutralize the system. Energy minimization of the solvated system was performed by using the steepest descent method. After minimization, a series of equilibrium MD simulations were performed: 200 ps of NVT and 1 ns of NPT. The time step of the MD simulation was set to 2 fs with the LINCS algorithm to constraint hydrogen-connected covalent bonds [57]. The long-range electrostatic interaction was calculated by the fast smooth particle-mesh Ewald (PME) electrostatics method [58,59]. At each step, v-rescale temperature coupling and Parrinello–Rahman pressure coupling were applied to maintain the system at 300 K. Lastly, MD simulations were conducted for 50 ns with a time step of 2 fs.

#### 4.3.2. Molecular Mechanics–Poisson Boltzmann Surface Area (MM-PBSA) Analysis

The binding free energies of the three hits were calculated with MM–PBSA analysis. We utilized the gmx_mmpbsa tool (https://github.com/Jerkwin/gmxtool/tree/master/gmx_mmpbsa, accessed on 10 August 2020) to analyze the binding free energy between each ligand and the RBPJ protein based on the trajectories obtained with GROMACS. For the dynamic trajectory of 50 ns, 50 frames were extracted at a time interval of 1000 ps and subsequently selected as input for MM–PBSA calculations. During the calculations, the binding free energy between ligand and RBPJ protein was decomposed into several terms: the gas-phase interaction energy (ΔE_MM_), non-polar solvation energy (ΔG_np_), polar solvation free energy (ΔG_pol_), and the conformational entropy (-TΔS). ΔE_MM_ generally contains coulomb electrostatic energy (ΔE_elec_) and van der Waals energy (ΔE_vdW_).

### 4.4. Cell Culture

The mouse breast cancer 4T1 cell line and human breast cancer MCF-7 cell line were purchased from the Type Culture Collection of the Chinese Academy of Sciences (Shanghai, China). The 4T1 cells were cultured in RPMI-1640 medium supplemented with 10% fetal bovine serum (FBS) and 1% penicillin/streptomycin, and the MCF-7 cells were cultured in DMEM supplemented with 10% FBS and 1% penicillin/streptomycin.

### 4.5. In Vitro Anticancer Activity

The 4T1 and MCf-7 cells were seeded in 96-well plates (5000 cells/well) in 5% CO_2_ at 37 °C and cultured for 24 h. Then, the cells were incubated with fresh media containing either fidaxomicin, schaftoside, or acarbose at various concentrations for 48 h. After further incubation with fresh media containing Cell Counting Kit-8 (CCK-8) solution for 2 h, the absorbance of each well was determined at 450 nm by a microplate reader.

### 4.6. Studies on the Mechanism of Anti-Tumor Action of Fidaxomicin

#### 4.6.1. Cellular Uptake and Intracellular Localization

The 4T1 cells were seeded in 15 mm confocal microscope dishes (2 × 10^4^ cells/dish) and incubated for one day. The stock fidaxomicin solution (53 mM) was diluted with complete RPMI-1640 medium to prepare working solutions (53 μM). The cells were incubated with working solutions for 0.5, 2, 4, 12, and 24 h. After incubation, cells were washed with PBS buffer and further fixed by using paraformaldehyde. Then, nuclei of all cells were stained with propidium iodide (PI) for about 10 min at 37 °C. Eventually, the dye solution was descanted, followed by washing three times with PBS and mounted with an anti-fluorescence quenching sealant.

The cells were then observed using a confocal laser scanning microscope (TCS SP5 II, Leica, Wetzlar, Germany). For fidaxomicin, the excitation wavelength was 351 nm, and the emission wavelength was 450 nm. For PI, the excitation wavelength was 488 nm, and the emission wavelength was 620 nm.

#### 4.6.2. Quantitative Real-Time PCR (qRT-PCR)

Cultured 4T1 cells were treated with fidaxomicin at a concentration of 32.3 μM for 24 h and 48 h. After washing with PBS buffer, total RNA was extracted from cells by using an RNA-Quick Purification Kit (ES-RN001, YiShan Biotech, Shanghai, China) and reverse-transcribed using a Fast All-in-One RT Kit (ES-RT001, YiShan Biotech, Shanghai, China). Quantitative PCR for mRNA was performed using qPCR Master Mix (ES-QP002, YiShan, Shanghai, China) according to the manufacturer’s protocol. The expression of mRNA was normalized to GAPDH. The primer sequences used in qRT-PCR are listed in Supplementary Material Table S3.

#### 4.6.3. Western Blot Assay

Cultured 4T1 cells were treated with fidaxomicin as mentioned above. After washing with PBS buffer, total cell extracts were prepared by using a Cell Total Protein Extraction Kit (Sangon Biotech, Shanghai, China) according to the manufacturer’s protocol. Protein concentration was measured by using a BCA kit. Each sample containing 2 mg/mL protein was separated by using sodium dodecyl sulfate–polyacrylamide gel electrophoresis (SDS–PAGE) and transferred onto a PVDF membrane, which was incubated overnight at 4 °C with primary antibodies. The primary antibodies used were rabbit anti mouse Hes1 (11988; Cell Signalling Technology, Danvers, MA, USA), rabbit anti mouse Hes5 (EPR15578; Abcam, Cambridge, UK), and rabbit anti mouse GAPDH (AF7021; Affinity Biosciences, Cincinnati, OH, USA). The membranes were next incubated with secondary antibodies (Goat Anti-Rabbit IgG (H + L) HRP, S0001; Affinity Biosciences) at room temperature for 2 h. According to the western blot chemiluminescence detection method, protein bands on the membrane were observed by using a chemiluminescent imaging system.

### 4.7. In Vivo Anti-Tumor Activity

Eight-week-old female BALB/c mice were purchased from the laboratory animal center of Jiangsu University. Animal studies were conducted under the guidelines of an approved protocol from the Institutional Animal Care and Use Committee at Jiangsu University (Protocol ID: 2021031202). To establish the tumor-bearing mouse model, 0.2 mL of 4T1 cell suspensions (2 × 10^7^ cells/mL) was inoculated to the mammary fat pad of BALB/c mice. When the tumor volume reached approximately 50–100 mm^3^, tumor-bearing mice were randomly assigned to six groups of five mice each. Fidaxomicin, DAPT, and 5-fluorouracil were first dissolved in DMSO as stock solutions and then administered after dilution with saline to achieve a final concentration of DMSO of less than 0.5%. Mice were treated with fidaxomicin-1 (50 mg/kg), fidaxomicin-2 (25 mg/kg), fidaxomicin-3 (5 mg/kg), DAPT (25 mg/kg), 5-Fluorouracil (25 mg/kg), and saline. Each formulation was injected intratumorally every other day. The anti-tumor efficiency was evaluated by monitoring the tumor volume. The tumor sizes were measured on two vertical axes using a caliper every other day and calculated as (width^2^ × length)/2. After 24 days, mice were sacrificed, and tumor tissues were excised and weighted.

The expressions of the Hes5 protein in tumor grafts were examined using Western blot assay. Total cell extracts were also prepared using a Cell Total Protein Extraction Kit (Sangon Biotech, Shanghai, China), as mentioned in the above Western blot method. The primary and secondary antibodies involved were the same as those used in the previous Western blot assay.

## 5. Conclusions

In summary, we identified fidaxomicin as a potential RBPJ-specific inhibitor through the use of a drug repurposing strategy. The antitumor activity of fidaxomicin was evaluated both in vitro and in vivo. It was shown that fidaxomicin could effectively inhibit the growth of two different breast cancer cell lines at micromolar concentrations. Furthermore, the administration of fidaxomicin resulted in a practical tumor inhibition in 4T1 tumor-bearing mice. Regarding the possible mechanism of action, the treatment of fidaxomicin lowered the expression of Notch downstream target genes at both transcriptional and translational levels, showing that fidaxomicin might potentially inhibit the formation of the RBPJ-dependent transcriptional complex. These results suggest that fidaxomicin might have potential for the treatment of RBPJ-dependent cancers. Additionally, this study represented an important step towards the repurposing of this FDA-approved drug for the treatment of breast cancer.

## Figures and Tables

**Table 1:** Energetic components of the binding energy for three hits in complex with RBPJ using MM-PBSA (kJ/mol).

Components	Fidaxomicin	Schaftoside	Acarbose
van der Waal energy (ΔE_vdW_)	−204.184	−182.001	−187.466
Electrostatic energy (ΔE_elec_)	−84.315	−106.519	−173.363
^a^ ΔE_MM_	−288.498	−288.52	−360.829
Polar solvation energy (ΔG_pol_)	141.176	210.695	307.811
Non-polar solvation energy (ΔG_np_)	−30.519	−24.261	−27.046
Configurational entropy (-TΔS)	101.286	58.367	126.046
Binding energy (^b^ ΔG_Bind_)	−76.555	−43.72	45.982

Note: ^a^ ΔE_MM_ = ΔE_vdW_ + ΔE_elec_, ^b^ ΔG_Bind_ = ΔE_MM_ + ΔG_pol_ + ΔG_np_ − TΔS.

## Data Availability

Data are contained within the article.

## Supplementary Materials

The following supporting information can be downloaded at: www.mdpi.com/article/10.3390/ph15050556/s1, Figure S1: The chemical structures of compounds used in this study; Figure S2: Schematic diagram of the interaction between Notch, MAML, DNA, and RBPJ; Table S1: Interactions between human transcription factor RBPJ and NICD; Table S2: Interactions between human transcription factor RBPJ and coactivator MAML; Table S3: Primer sequence.
