# Supplementary material for: Identification of Potential RBPJ-Specific Inhibitors for Blocking Notch Signaling in Breast Cancer Using a Drug Repurposing Strategy
Source: Pharmaceuticals (Basel). 2022 Apr 29;15(5):556. doi: 10.3390/ph15050556 (PMC9146688; doi:10.3390/ph15050556)
Supplement: Supplementary file 1 [file pharmaceuticals-15-00556-s001.zip › pharmaceuticals-1662583-supplementary.pdf]

# Identification of RBPJ-specific inhibitors for blocking Notch signaling in breast cancer using drug repurposing strategy

**Mengjie Rui[1] , Min Cai[1], Yu Zhou[1], Wen Zhang[1], Lianglai Gao[1], Ke Mi[1], Wei Ji[1], Dan Wang[1], Chumley Feng[1,*]**

[1] Department of Pharmaceutics, School of Pharmacy, Jiangsu University, Zhenjiang 212013, PR China; mjrui@ujs.edu.cn (M. R.); 2211915001@stmail.ujs.edu.cn (M. C.); 2221815020@stmail.ujs.edu.cn (Y. Z.); 2221915025@stmail.ujs.edu.cn (W. Z.); 2222015007@stmail.ujs.edu.cn (L. G.); 2212015010@stmail.ujs.edu.cn (K. M.); jinjian@ujs.edu.cn (W. J.); danwang@ujs.edu.cn (D. W.); feng@ujs.edu.cn (C. F.)

*Correspondence: feng@ujs.edu.cn;

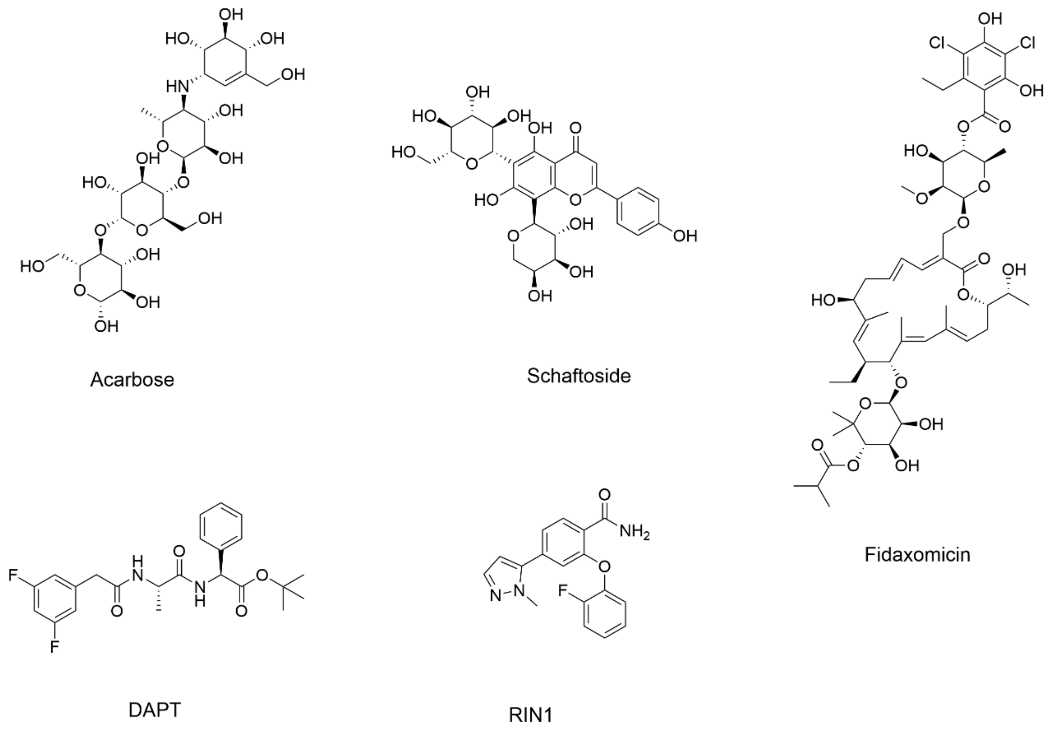


Figure S1. The chemical structures of compounds used in this study.

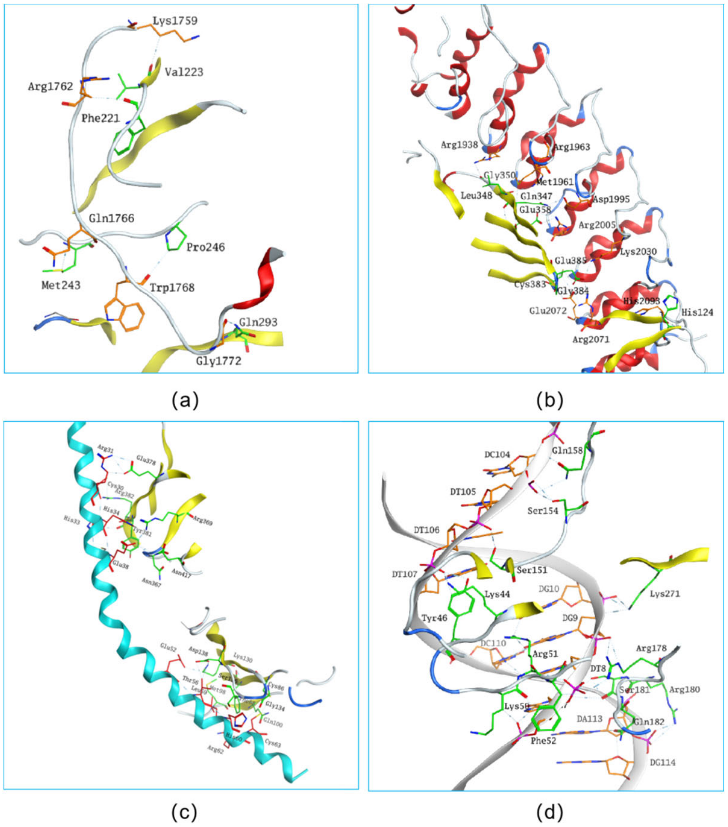


Figure S2. Schematic diagram of the interaction between Notch, MAML, DNA and RBPJ. (a) shows the schematic diagram of the interaction between RAM domain and RBPJ in Notch receptor. (b) shows the schematic diagram of the interaction between the ANK domain and RBPJ in the Notch receptor. (c) shows the schematic diagram of the interaction between DNA and RBPJ. (d) represents the schematic diagram of the interaction between MAML and RBPJ.

Table S1 Interactions between human transcription factor RBPJ and NICD

| Residues of NICD | Residues of RBPJ | Interactions |
|---|---|---|
| Lys1759 | Val223 | H-bond |
| Arg1762 | Phe221 | H-bond |
| Gln1766 | Met243 | H-bond |
| Trp1768 | Pro246 | H-bond |
| Gly1772 | Gln293 | H-bond |
| Arg1938 | Gly350 | H-bond |

| | | |
|---|---|---|
| Met1961、Met1993、Asp1995 | Gln347 | H-bond |
| Arg1963 | Leu348 | H-bond |
| Arg2005 | Glu358 | H-bond |
| Lys2030 | Glu385 | H-bond |
| Glu2072 | Cys383、Gly384 | H-bond |
| His2093、Met2094 | His124 | H-bond |

Table S2 Interactions between human transcription factor RBPJ and coactivator MAML

| Residues of MAML | Residues of RBPJ | Interactions |
|---|---|---|
| Cys30 | Arg382 | H-bond |
| Arg31 | Arg378 | H-bond |
| His33 | Arg382 | H-bond |
| His34 | Tyr381 | H-bond |
| Glu38 | Asn367、Arg369、Asn417 | H-bond |
| Glu52 | Lys130 | H-bond |
| Thr56 | Lys130、Asp138 | H-bond |
| Leu59 | Phe88 | p-π conjugation |
| His60 | Gly134、Ser136 | H-bond、p-π conjugation |
| Arg62 | Met98 | H-bond |
| Cys63 | Cys86 | H-bond |

Table S3 Primer sequence

| **Primer** | **Sequences (5' to 3')** |
|---|---|
| GAPDH-F | GGTTGTCTCCTGCGACTTCA |
| GAPDH-R | TGGTCCAGGGTTTCTTACTCC |
| Notch1-F | AGACCAACATCAATGAGTGCCACAG |
| Notch1-R | GCATAAGCAGAGGTAGGAGTTGTCAC |
| Hes1-F | GAGGCGAAGGGCAAGAATAAATGAAAG |
| Hes1-R | AATGTCTGCCTTCTCTAGCTTGGAATG |
| Hes5-F | GCATGTGGGCACGATTTTGTACTTAG |
| Hes5-R | ATTAGAAGCCTTCAGAACAGCCTGTG |

| | |
|---|---|
| Hey1-F | GAAAGGTGTCTGTGCCCTGAATCC |
| Hey1-R | CATAACTGTCTTGCTTGCTGCCAAC |
